# Neuromuscular recovery from botulism involves multiple forms of compensatory plasticity

**DOI:** 10.3389/fncel.2023.1226194

**Published:** 2023-08-15

**Authors:** James B. Machamer, Edwin J. Vazquez-Cintron, Mallory J. Stenslik, Kathleen T. Pagarigan, Aaron B. Bradford, Celinia A. Ondeck, Patrick M. McNutt

**Affiliations:** ^1^BASF, Research Triangle Park, NC, United States; ^2^United States Army Medical Research Institute of Chemical Defense, Gunpowder, MD, United States; ^3^Wake Forest Institute for Regenerative Medicine, Wake Forest University School of Medicine, Winston-Salem, NC, United States

**Keywords:** botulinum neurotoxin, mice, endplate recordings, voltage-gated calcium channels, compensatory plasticity, hemidiaphragm:phrenic nerve preparations, twitch and tetanic contractions, running wheel activity

## Abstract

**Introduction:**

Botulinum neurotoxin (BoNT) causes neuroparalytic disease and death by blocking neuromuscular transmission. There are no specific therapies for clinical botulism and the only treatment option is supportive care until neuromuscular function spontaneously recovers, which can take weeks or months after exposure. The highly specialized neuromuscular junction (NMJ) between phrenic motor neurons and diaphragm muscle fibers is the main clinical target of BoNT. Due to the difficulty in eliciting respiratory paralysis without a high mortality rate, few studies have characterized the neurophysiological mechanisms involved in diaphragm recovery from intoxication. Here, we develop a mouse model of botulism that involves partial paralysis of respiratory muscles with low mortality rates, allowing for longitudinal analysis of recovery.

**Methods and results:**

Mice challenged by systemic administration of 0.7 LD_50_ BoNT/A developed physiological signs of botulism, such as respiratory depression and reduced voluntary running activity, that persisted for an average of 8–12 d. Studies in isolated hemidiaphragm preparations from intoxicated mice revealed profound reductions in nerve-elicited, tetanic and twitch muscle contraction strengths that recovered to baseline 21 d after intoxication. Despite apparent functional recovery, neurophysiological parameters remained depressed for 28 d, including end plate potential (EPP) amplitude, EPP success rate, quantal content (QC), and miniature EPP (mEPP) frequency. However, QC recovered more quickly than mEPP frequency, which could explain the discrepancy between muscle function studies and neurophysiological recordings. Hypothesizing that differential modulation of voltage-gated calcium channels (VGCC) contributed to the uncoupling of QC from mEPP frequency, pharmacological inhibition studies were used to study the contributions of different VGCCs to neurophysiological function. We found that N-type VGCC and P/Q-type VGCC partially restored QC but not mEPP frequency during recovery from paralysis, potentially explaining the accelerated recovery of evoked release versus spontaneous release. We identified additional changes that presumably compensate for reduced acetylcholine release during recovery, including increased depolarization of muscle fiber resting membrane potential and increased quantal size.

**Discussion:**

In addition to identifying multiple forms of compensatory plasticity that occur in response to reduced NMJ function, it is expected that insights into the molecular mechanisms involved in recovery from neuromuscular paralysis will support new host-targeted treatments for multiple neuromuscular diseases.

## 1. Introduction

Botulinum neurotoxins (BoNTs) are bacterial toxins responsible for the neuroparalytic disease of botulism. BoNTs are highly potent toxins, with estimated human lethal doses ranging from 0.1 to 100 ng/kg, depending on the route of exposure. Clinical botulism can result from ingestion, inhalation or injection of preformed toxin, or following infection with toxin-producing strains of *Clostridium* (Pirazzini et al., 2017). Although botulism is rare in humans, mass casualty events can be caused by accidental or deliberate exposure to BoNT (Wein and Liu, 2005; Villar et al., 2006). The widespread availability of BoNT-producing bacterial strains, extreme lethality, and lack of specific treatments have resulted in classification of BoNTs as Tier 1 select agents, which are at greatest risk of deliberate misuse with most significant potential for mass casualties (Arnon et al., 2001).

Botulinum neurotoxins cause prolonged but reversible muscle denervation without direct trauma to motor neurons. Flaccid muscle paralysis results from a complex set of molecular actions, involving selective uptake into motor neurons, translocation into the cytosol, and proteolytic cleavage of presynaptic proteins necessary for synaptic exocytosis (Simpson, 2004). The active form of the neurotoxin is a heterodimer protein consisting of a 100 kDa heavy chain (HC) and 50 kDa light chain (LC) that remain attached through electrostatic interactions and a single disulfide bond (Pirazzini et al., 2017). The C-terminal of HC mediates highly selective and efficient binding to endosomal and ganglioside receptors on the presynaptic membrane of peripheral neurons (Bandyopadhyay et al., 1987). Following neuronal uptake via endocytosis, the N-terminal domain of HC facilitates translocation of LC across the endosomal membrane to the presynaptic cytosol (Shone et al., 1987; Fischer and Montal, 2007; Winner et al., 2020), where LC specifically targets and cleaves SNARE proteins essential for the fusion of synaptic vesicles. As the concentration of cleaved SNARE proteins at the presynaptic membrane increases, the motor nerve terminals cannot reliably elicit muscle contraction, causing muscle weakness that progresses to flaccid paralysis (Bradford et al., 2018). Some BoNT serotypes cause muscle paralysis that can last for weeks or longer, triggering secondary morphological changes, such as NMJ degeneration and muscle atrophy, which can contribute to delayed recovery (Borodic et al., 1994; Williams et al., 2013).

Clinical symptoms of botulism emerge 12–36 h after exposure to BoNT, resulting from peripheral blockade of neurotransmission at neuromuscular junctions (NMJs) and autonomic nerve terminals (Sobel, 2005). At lethal doses, toxic signs typically include generalized muscle weakness, descending flaccid paralysis, and death from respiratory failure. Post-exposure prophylaxis with equine-derived antitoxin can prevent botulism symptoms, but only if given shortly after exposure. Approximately 70% of botulism patients given antitoxin within 48 h after symptomatic emergence still require mechanical ventilation for survival (Yu et al., 2017; Richardson et al., 2019). Once botulism symptoms develop, survival from a lethal exposure requires sustained administration of mechanical ventilation until natural recovery of respiratory function. Respiratory paralysis can continue for weeks or longer, requiring chronic ventilation with an increased risk of life-threatening comorbidities (Sobel, 2005; Touman and Stratakos, 2018).

Neuromuscular junctions are specialized synapses that exhibit robust forms of short-term and long-term plasticity (Ouanounou et al., 2016). Although neuromuscular junctions between phrenic motor neurons and diaphragm nerve fibers are the main clinical targets of botulism (Sobel, 2005), few studies have characterized neurophysiological mechanisms of diaphragm intoxication and recovery from intoxication. This is mainly due to the difficulty in administering doses of BoNT that cause respiratory toxic signs without also causing respiratory failure (Emanuel et al., 2019). Although isolated diaphragm muscle preparations have been used to characterize the acute effects of BoNT intoxication on phrenic neurotransmission, the short useful life of these preparations (6–8 h) precludes their use in studying longer-term responses *ex vivo* (Stanley and Drachman, 1983; Dolly et al., 1987; Bradford et al., 2018; Vazquez-Cintron et al., 2020). Alternatively, studies to understand mechanisms of paralysis and recovery have mainly focused on local administration of paralytic doses of BoNT to skeletal muscles of the limb followed by functional characterization over time (Morbiato et al., 2007; Rogozhin et al., 2008). However, diaphragm fibers have specialized features of excitation- contraction coupling that are mediated, in part, through expression of the diaphragm-specific RyR3 isoform of the sarcoplasmic reticulum calcium release channel (Conklin et al., 2000; Rossi et al., 2001). Diaphragm muscles are highly specialized to contract continuously and rhythmically (Polla et al., 2004) with fibers that are functionally and structurally distinct from limb skeletal muscle (Hughes and Whaler, 1962; Stuelsatz et al., 2012). Diaphragm muscle fibers exhibit relatively small training-induced changes in aerobic capacity or fiber type (Dempsey, 1986; Powers et al., 2011) and atrophy develops slowly in paralyzed diaphragm muscles, manifesting over weeks to months vs. days in paralyzed limb skeletal muscles (Welvaart et al., 2011). These differences suggest the response of diaphragm muscles to reduced activity following BoNT intoxication may be mechanistically distinct from limb skeletal muscle.

Here we characterized longitudinal changes in neuromuscular transmission at diaphragm neuromuscular junctions (NMJs) following systemic intoxication with sub-lethal doses of BoNT/A. We chose to use BoNT/A in these studies for several reasons. BoNT/A is responsible for approximately half of natural botulism cases in the United States and thus represents an important clinical target (Centers for Disease Control and Prevention [CDC], and U. S. Department of Health and Human Services, 2021). BoNT/A is also the most persistent serotype, producing long-lasting paralytic effects that are amenable to long-term neurophysiological assessments (Foran et al., 2003). Finally, BoNT/A is the active component in most BoNT-based pharmaceuticals (Brashear, 2008). We anticipate that characterization of endogenous mechanisms of neurophysiological recovery from BoNT/A paralysis will inform treatment strategies for botulism symptoms caused by natural or iatrogenic exposures. Mice challenged by systemic administration of BoNT/A developed physiological, functional and neurophysiological evidence of respiratory paralysis. Recovery of diaphragm muscle function preceded recovery of neurotransmission in individual end plates, suggesting a role for compensatory processes in response to low-amplitude synaptic transmission. Multiple forms of synaptic plasticity were identified that increased the probability of muscle contraction, providing a mechanism for uncoupling of end plate recovery from physiological recovery. These studies illustrate several compensatory mechanisms activated in response to BoNT/A paralysis, including modulation of the Ca^2+^ sensitivity of evoked neurotransmission. Furthermore, they identify endogenous mechanisms that may be targeted pharmacologically to treat diaphragm weakness caused by botulism and other neuromuscular diseases.

## 2. Materials and methods

### 2.1. Animal use and husbandry

All procedures were conducted in accordance with the principles stated in the Guide for the Care and Use of Laboratory Animals and the Animal Welfare Act of 1,966 (P.L. 89–544), and reported in accordance with ARRIVE 2.0 guidelines. For all studies, female CD-1 mice (6–10 weeks; RRID:IMSR_CRL:022, Charles River Laboratories, Wilmington, MA, USA) weighing 25–32 g were individually housed at 20–23°C on a 12:12 h light:dark cycle and provided a standard diet with regular enrichment and water *ad libitum*. Mice were given unconstrained access to running wheels for 12 days prior to start of experiments. For *ex vivo* studies, mice were anesthetized using 5% isoflurane and euthanized by decapitation prior to phrenic nerve-hemidiaphragm dissection.

### 2.2. Determination of BoNT/A LD_50_

Botulinum neurotoxins serotype A1 was purchased from Metabiologics Inc. (lot A0818916; Madison, WI, USA) at 10 μg/mL and stored at 4°C. The specific activity was determined using the mouse lethal bioassay (Pearce et al., 1994). Briefly, BoNT/A was diluted in gel phosphate buffer (GPB; 0.2% gelatin in PBS, pH 7.4) to reduce the absorption of toxin to plastic surfaces prior to injection. Mice were challenged by intraperitoneal injection with BoNT/A at 0.10, 0.125, 0.150, 0.175, 0.20, 0.225, 0.250, and 0.30 pg/g (*n* = 4–8 per group) and survival rates were monitored for 4 days. The median lethal dose was estimated to be 0.17 ng/kg (95% CI: 0.14–0.20 ng/kg) from simple logistical regression of survival outcomes (Supplementary Figure 1).

### 2.3. Assessment of toxic signs

Treatments were given between 4 and 6 PM, prior to the start of nightly running. Mice were intoxicated by intraperitoneal (i.p.) challenge with 0.095 ng/kg (0.55 LD_50_), 0.120 ng/kg (0.7 LD_50_) or 0.145 ng/kg (0.85 LD_50_) BoNT/A prepared in 0.25 mL GPB and administered using a 0.30 mL glass Hamilton syringe (Reno, NV, USA) and 18-1/2 gauge disposable needles. In some cases, 0.7 LD_50_ BoNT/A was mixed with 1,000-fold molar excess sheep anti-BoNT/A antitoxin (generous gift of Charles Shoemaker, Tufts University) prior to injection. Mice were given warm 2% sucrose-lactate Ringer’s solution (20 mL/kg, subcutaneous) once daily from 3 to 7 days after intoxication to mitigate dehydration. Mice were scored for signs of respiratory botulism daily using a toxic signs (TS) scoring system modified to focus on respiratory signs: 0 = no visible signs of intoxication, 1 = paradoxical abdominal breathing or 2 = forced agonal breathing (Vazquez-Cintron et al., 2020; Machamer et al., 2022). The proportion of mice exhibiting toxic signs were compared using chi-square analysis in Graphpad Prism v9 (Graphpad Software, La Jolla, CA, USA).

### 2.4. Running wheel activity

The number of running wheel rotations between 6 PM and 6 AM was measured using Hall sensors attached to a 16-station home cage running wheel system (Columbus Instruments). The distance run was calculated by multiplying the running wheel inner diameter by the number of rotations in 5 min intervals. Mice were acclimated to running wheels for 12 days prior to intoxication, which was sufficient to produce stable levels of nightly running activity. Nightly running distances were normalized to the average distance run during the three nights prior to BoNT/A injection. Mice were challenged with 0.7 LD_50_ BoNT/A with or without 1,000-fold molar excess of antitoxin. Raw running wheel data were exported as.csv files and processed for visualization and statistical analysis using a custom python script. Statistical significance was determined by two-way ANOVA with Dunnett’s multiple comparisons test (comparing running wheel activity post-injections to baseline activity) in Graphpad Prism v9.

### 2.5. Assessment of twitch and tetanic contraction strengths

Diaphragms were dissected from mice anesthetized with isoflurane and euthanized by decapitation at 3, 7, 14, 21, or 28 days after intraperitoneal challenge with 0.7 LD_50_ BoNT/A. Full diaphragms, associated ribs, intercostal muscles, and connected phrenic nerve were isolated and hemisected in room temperature, oxygenated (95% O_2_/5% CO_2_) Tyrode’s solution (in mM: 137 NaCl, 5 KCl, 1.8 CaCl_2_, 1 MgSO_4_, 24 NaHCO_3_, 1 NaH_2_PO_4_, and 11 D-glucose, pH 7.4) to produce two phrenic nerve-hemidiaphragms per mouse. Excess tissues not directly involved in muscle contraction were trimmed from the ribs, diaphragm muscle, and nerve. Fascia was carefully removed from the nerve fiber to ensure reproducible stimulation. Prior to loading tissue into the organ baths, Tyrode’s solution was warmed to 37°C and oxygenated by bubbling with 95% O_2_/5% CO_2_ (Radnoti LLC, Covina, CA, USA). A metal hook was impaled centrally along the circumference of the diaphragm muscle through the intercostal muscle of the ribs, and silk cord was tied in a double knot around the crural diaphragm or the tendon that separates the muscles. Another silk cord was tied to the severed end of the phrenic nerve to mount the phrenic nerve into the stimulating electrode. The hemidiaphragm was immersed in the tissue bath, and the metal hook was used to anchor the ribcage to a glass hook at the bottom of the bath, while silk cord tied to the crural diaphragm was attached to an isometric force transducer above the bath. Once securely hooked and tied, the tension of the muscle was adjusted to resting tension (0.5 g). The nerve suture was carefully threaded through custom stainless steel, bipolar loop electrodes (Radnoti LLC), and the nerve bundle itself laid on the metal of the electrode while the suture knot held the tissue in place. The nerve and electrode were angled away from the tissue to prevent direct stimulation of muscle fibers.

Hemidiaphragms were allowed to acclimate to temperature and oxygenation with 0.05 Hz twitch stimulation for at least 30 min. Hemidiaphragms were stimulated and recorded in groups of 4 using PowerLab stimulating/recording hardware and LabChart software (AD Instruments, Dunedin, New Zealand). Raw tension was filtered with a digital low-pass filter of 50 Hz to eliminate high-frequency noise. The cyclical measurement feature was used to analyze contraction tension peak height, baseline tension, and normalized peak heights. After determination of optimal length and supramaximal stimulation, baseline twitch strength was determined from at least 5 min of recording. For tetanic stimulation, three trains of fifty 0.2 ms square pulses were applied to hemidiaphragms, with 1 min intervals between trains. Stimulations were provided at 1, 25, 50, 60 70, 80, 90, 100, 125, 150, 175, 200, and 250 Hz. The maximum contraction strength was recorded after each stimulation and averaged among the three stimulations at each frequency. The half maximal frequency was calculated as the frequency of stimulation that resulted in half the maximum contraction strength, determined from curve fitting using non-linear regressions in Graphpad Prism. Statistical significances were determined using one-way ANOVA with Dunnett’s multiple comparisons test.

### 2.6. Electrophysiological recordings

Hemidiaphragms were dissected as above and gently stretched and pinned with stainless steel insect pins in 3.5 cm diameter tissue culture plates filled with Sylgard 184 silicone elastomer (Sigma-Aldrich) and oxygenated Tyrode’s. Functional viability of hemidiaphragm was confirmed by stimulating the phrenic nerve with a parallel bipolar stimulation electrode (FHC, Bowdoin, ME, USA) attached to a MM33 manual micromanipulator (Marshauser Weltzer, Wetzlar, Germany). The phrenic nerve was positioned between the two leads, and stimulation was delivered by single suprathreshold 0.2 ms pulse from a connected DS3 voltage isolation unit (Digitimer, Hertfordshire, United Kingdom). Mu-conotoxin GIIIB (1 μM; Alomone labs, Jerusalem, Israel) in Tyrode’s solution was added and muscle contraction was monitored as low frequency (0.2 Hz) suprathreshold stimuli were delivered. After diaphragms were fully paralyzed, serial recordings were performed from individual muscle fibers throughout the entire diaphragm at room temperature. Recordings were made using a 10–15 MΩ sharp electrode filled 3 M KCl attached to HEKA Patchmaster (HEKA Instruments, Holliston, MA, USA) in current clamp mode (0 mA). Recording electrodes were inserted into muscle fibers near the end plates using a dissecting stereo microscope. Oxygenated Tyrode’s solution was exchanged every hour until experiments were complete (up to 6 h).

### 2.7. Analysis of end plate recovery

For each muscle fiber, the resting membrane potential (RMP) was recorded within 1 min of establishing a viable recording. The amplitude and success rate of end plate potentials (EPPs) were measured by delivering 10 stimuli to the phrenic nerve at 0.2 Hz. Miniature EPPs (mEPPs) were measured during 2 min of spontaneous activity. EPP amplitudes were analyzed using Axograph X (AxoGraph Scientific). mEPPs were detected using a template-based search algorithm in Axograph X. Because BoNT intoxication altered the shapes of the mEPPs, a different template was used for each different intoxication condition (days post-intoxication/control). An initial template generated from control endplates was used to detect a preliminary set of mEPPs from ∼5 different endplates. These mEPP traces were then used to form a new template that was used for the final round of mEPP detection. Due to the larger variability in the kinetics of the mEPP, the variables chosen by the search algorithm to minimize false negatives resulted in false positives, which were identified by eye and eliminated by hand. Quantal content (QC) for each endplate was calculated by dividing the average EPP amplitude by the average mEPP amplitude and correcting for non-linear summation (McLachlan and Martin, 1981). For endplates with fewer than 10 mEPPs, the average mEPP amplitude of all muscle fibers in the given recording condition was used to calculate quantal content. Statistical significance was determined using one-way analysis of variance (ANOVA) with Dunnett’s multiple comparison test for mEPP amplitude, mEPP half width and RMP; aand Kruskal-Wallis test with Dunn’s multiple comparisons test for success rates, EPP amplitude, quantal content (QC), and mEPP frequency. Recovery of QC and mEPP frequencies at 28 days after intoxication was compared using Mann-Whitney test for non-normal distributions.

### 2.8. Analysis of calcium channel blockers

Experiments were performed on hemidiaphragms from mice challenged with vehicle or BoNT/A at 21 days post-treatment. Mice had unrestrained access to running wheels throughout the study. For each hemidiaphragm, baseline recordings of EPP success rate, EPP amplitude and mEPP frequency were made from 20 separate muscle fibers. Hemidiaphragm preparations were then incubated with vehicle (Tyrode’s solution), 10 μM nimodipine (Tocris Bioscience, Minneapolis, MN, USA), 1 μM ω-conotoxin GVIA (Alomone labs) or 0.2 μM ω-agatoxin IVA (Alomone labs) for 1 h and recordings were repeated for an additional 20 muscle fibers.

## 3. Results

**FIGURE 1:**
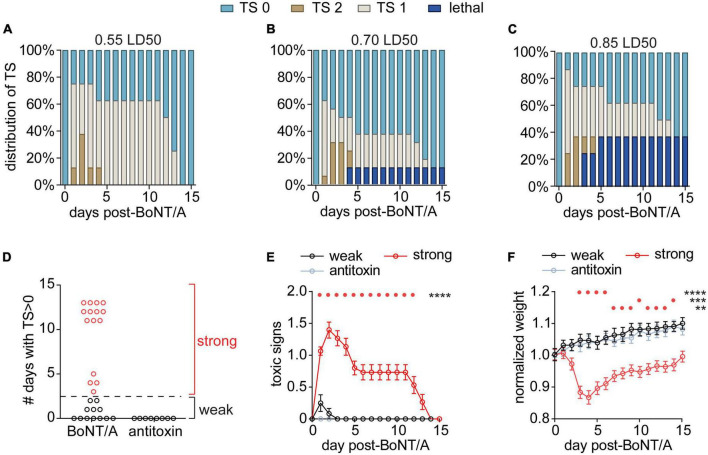
Establishing a model of systemic botulism with respiratory signs. Mice were challenged by intraperitoneal injection of 0.55 LD_50_ (*n* = 8), 0.70 LD_50_ (*n* = 16), or 0.85 LD_50_ (*n* = 8) BoNT/A and the respiratory toxic signs (TS) were scored daily. Mice with no clinical signs of botulism were scored TS 0, mice displaying paradoxical abdominal breathing (wasp waist) were scored TS 1, and mice exhibiting forced agonal breathing were scored TS 2. **(A–C)** Distribution of TS scores and survival outcomes for each BoNT dose. **(D)** The number of days that surviving mice remained symptomatic (TS > 0; *n* = 27) independent of dose. Mice in which toxic signs resolved to 0 within 3 days were grouped into the “weak responder” group (*n* = 12), while mice in which toxic signs resolved by 4 days or later were grouped into the “strong responder” group (*n* = 15). **(E)** Toxic signs were monitored among strong responders, weak responders and antitoxin-treated mice (*n* = 8) until fully resolved and compared using two-way RM ANOVA [*F*_(30,480)_ = 16.9; *p* = < 0.0001]. Daily toxic signs were compared to antitoxin-treated mice using Dunnett’s multiple comparisons test and significant differences are depicted above the graph. **(F)** Weight changes were monitored among strong responders, weak responders and antitoxin-treated mice and compared using two-way RM ANOVA [*F*_(30,480)_ = 14.9; *p* = < 0.0001]. Daily weights were compared to antitoxin-treated mice using Dunnett’s multiple comparisons test and significant differences are depicted above the graph. For all panels, ****p < 0.0001, ***p < 0.001, **p < 0.01.

With the goal of developing a sublethal model of systemic botulism, mice were challenged by intraperitoneal administration of 0.55 LD_50_ (*n* = 8), 0.70 LD_50_ (*n* = 16), or 0.85 LD_50_ (*n* = 8) BoNT/A and toxic signs were monitored over time (Figures 1A–C; Vazquez-Cintron et al., 2020). Intoxication produced respiratory signs of botulism, including abdominal paradox and altered respiratory pattern, in 75% (0.55 LD_50_), 62.5% (0.7 LD_50_), and 87.5% (0.85 LD_50_) of mice, respectively. There were no significant differences in the overall proportion of mice that developed toxic signs among the three doses (χ^2^ = 1.70, *p* = 0.43) nor in the proportion that progressed to mild (χ^2^ = 0.13 *p* = 0.94) or severe toxic signs (χ^2^ = 0.14, *p* = 0.93). There was a trend toward increasing mortality at higher toxin doses, however, differences in mortality were not significant at these group sizes (χ^2^ = 4.5, *p* = 0.11). Collectively these data suggest that 0.55–0.85 LD_50_ produce generally similar outcomes in terms of toxic presentation, albeit with a trend towards increased mortality at higher doses.

Among the different challenge doses, surviving mice could be classified into two groups based on the duration of respiratory signs: strong responders (*n* = 15) exhibited respiratory signs lasting ≥3 days after injection, whereas weak responders (*n* = 12) developed respiratory signs that resolved within 3 days or did not develop respiratory signs at all (Figure 1D). There were no apparent differences among doses in duration of toxic signs (χ^2^ = 1.63 *p* = 0.44) so the different doses were aggregated for subsequent analysis. Strong responders exhibited toxic signs for an average of 10 days (95% CI: 8–12 days; Figure 1E) and underwent acute weight loss after intoxication (Figure 1F). Weak responders exhibited transient symptoms of botulism that rapidly resolved without weight loss (Figures 1E, F). In comparison, control mice challenged with BoNT/A plus antitoxin exhibited neither toxic signs of botulism nor weight loss, confirming toxic signs resulted from BoNT toxemia (Figures 1E, F). Given the lack of difference among the challenge doses, subsequent studies used exclusively 0.7 LD_50_ BoNT/A challenge.

Voluntary running activity has been used to monitor the physiological effects of intoxication in mice (Keller, 2006; Kutschenko et al., 2016; Schwartz et al., 2021). To further interrogate the apparent differences between strong and weak responders, mice were challenged with BoNT/A or BoNT/A plus antitoxin and nocturnal running activity was monitored for 20 days (Supplementary Figure 2). Weak responders (*n* = 24) and antitoxin-treated mice (*n* = 8) showed no reductions in running activity. In strong responders (*n* = 26), running distances declined by 85.8 ± 4.1% within 2 days after injection and gradually returned to baseline over the next 2 weeks, paralleling changes in toxic signs (Supplementary Figures 2A–G). Strong responders exhibited shorter distances per running bout (Supplementary Figure 2H) and fewer bouts per night (Supplementary Figure 2I), consistent with reduced respiratory capacity and/or skeletal muscle weakness (Amann, 2012). Taken together, these results suggested strong responders suffered systemic botulism with respiratory involvement. Further studies focused on strong responders, as these mice exhibited clinical and physiological evidence of severe respiratory toxemia.

**FIGURE 2:**
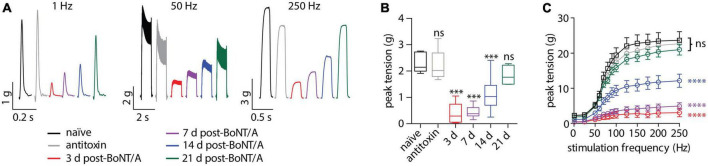
Diaphragm function is impaired in strong responders for over 14 days after BoNT/A injection. Twitch and tetanic muscle contractions were elicited from hemidiaphragms removed from mice given vehicle, 0.7 LD_50_ BoNT/A or 0.7 LD_50_ BoNT/A plus antitoxin. **(A)** Representative *ex vivo* muscle tension traces resulting from 1 Hz (twitch), 50 or 250 Hz stimulation. **(B)** Average contraction strength from a single superthreshold 0.2 ms stimulus (twitch). Statistical significances vs. vehicle were determined by a one-way ANOVA with Dunnett’s post-test. **(C)** Force-frequency plots produced by stimulating the phrenic nerve from 25 to 250 Hz (*n* = 6 per group). No differences among groups were apparent (*p* = 0.08). Statistical significances among groups were determined using an extra sum-of-squares *F*-test on median force-frequency values determined from four-parameter variable slope non-linear fit. ^***^p < 0.001, ^****^p < 0.0001.

To directly confirm respiratory weakness in strong responders, nerve-elicited contraction strengths were measured in phrenic nerve-hemidiaphragm preparations isolated from naïve mice, strong responders and mice treated with BoNT/A plus antitoxin. In strong responders, twitch contraction strengths significantly declined to 17.3 ± 18.0% of naïve values at 3 days after intoxication and progressively recovered to baseline between 7 and 21 days (Figures 2A, B). Longitudinal changes in sub-tetanic and tetanic muscle contraction strengths closely paralleled twitch contractions, with significant decreases in contraction strength that recovered to baseline by 21 days (Figures 2A, C). No differences were apparent between naïve diaphragms and diaphragms from antitoxin-treated mice (*p* = 0.99). The half-maximal force-frequency was not significantly changed during the recovery from intoxication [*F*_(5,29)_ = 2.1; *p* = 0.09, one-way ANOVA; Table 1], suggesting BoNT/A intoxication does not affect use-dependent facilitation of diaphragm muscle contraction.

**TABLE 1:** Summary of force-frequency measurements.

	Naïve	Antitoxin	3 days	7 days	14 days	21 days
Median frequency (Hz)	72.58	74.28	69.83	74.85	73.61	74.61
95% CI	71.2–73.4	72.6–76.0	65.8–73.8	72.1–77.6	70.6–76.6	73.2–76.0
*R* ^2^	0.99	0.99	0.96	0.98	0.96	0.99

**FIGURE 3:**
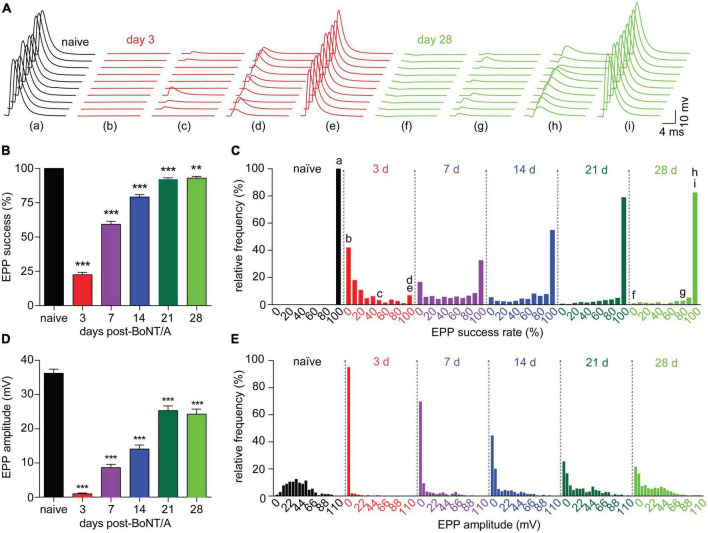
Diaphragm end plates remain intoxicated for weeks after a sublethal BoNT/A intoxication. Neurophysiological properties were evaluated in diaphragms removed from naïve mice and strong responders at 3, 7, 14, and 21, and 28 days after IP injection of 0.7 LD_50_ BoNT/A. **(A)** Representative sets of 10 EPPs recorded in end plates from naïve, 3 or 28 days post-BoNT/A mice illustrate the high degree of variability observed in end plates from intoxicated diaphragms both early and late in recovery. Lower case letters correspond to the indicated histogram bin in **(C)**. **(B)** The average EPP success rate (percentage of stimuli resulting in a measurable EPP) is significantly reduced after BoNT/A injection and fails to recover through 28 days. **(C)** Frequency distribution of EPP success rate per end plate demonstrates partial recovery from intoxication through 28 days. **(D)** Average EPP amplitudes were reduced at 3, 7, 14, 21, and 28 days after intoxication. **(E)** Frequency distribution reveals a bimodal recovery of EPP amplitudes, with fully intoxicated end plates persisting through 28 days. For success rate and EPP amplitude: *N* = 7 naïve mice and 249 end plates; for 3 days post-BoNT/A, *N* = 7 mice and 278 end plates; for 7 days post-BoNT/A, *N* = 7 mice and 323 end plates; for 14 days post-BoNT/A, *N* = 6 mice and 297 end plates; for 21 days post-BoNT/A, *N* = 7 mice and 355 end plates; and for 28 days post-BoNT/A, *N* = 6 mice and 256 end plates. Data are plotted as mean and S.D. ***p < 0.001, **p < 0.01. Statistical significance vs. naïve end plates was determined using the Kruskal-Wallis test with Dunn’s post-test for success rates and EPP amplitudes and using one-way ANOVA with Dunnett’s post-test for mEPP amplitudes and RMPs.

Because muscle fiber contraction is an “all-or-none” response to threshold release of acetylcholine. measurements of contraction strength can obscure graded changes in neurotransmission. To characterize neurophysiological changes during recovery from intoxication, nerve-evoked end plate potentials (EPPs) were recorded in diaphragms isolated from naïve mice and strong responders at 3, 7, 14, 21, and 28 days after BoNT/A injection. In naïve end plates, a large EPP was observed after each stimulus of a ten-stimulus train (0.2 Hz), producing an EPP success rate of 100% (Figures 3A–C). By 3 days after intoxication, the EPP success rate declined to 22.5%, with 93.2% of end plates suffering one or more stimulation failures per train. Although success rates progressively improved over time, they remained significantly depressed at 28 days, with 17.5% of end plates exhibiting at least one neurotransmission failure. Consistent with decreased success rate, EPP amplitude was reduced to 2.8% of naïve end plates by 3 days after intoxication and remained significantly depressed through 28 days (Figures 3D, E). EPPs were infrequent at early time points (Figure 3C) and, when present, usually involved ≤2 quanta (Figure 3E). Histogram analysis of EPP amplitudes revealed a subpopulation of end plates that remained stubbornly intoxicated through 28 days (Figure 3E). Incidentally, we also observed the significant depolarization of muscle fiber resting membrane potential at 3, 7, and 14 days (Supplementary Figures 3A, B), which may allow muscle fibers to contract more readily in response to decreased acetylcholine release. These data reveal that recovery of neurotransmission remains incomplete at 28 days, despite the apparent reversal of respiratory toxemia and restoration of diaphragm muscle contraction strengths (Figure 2). Furthermore, they illustrate multiple compensatory mechanisms that are activated in the NMJ during the recovery from intoxication.

**FIGURE 4:**
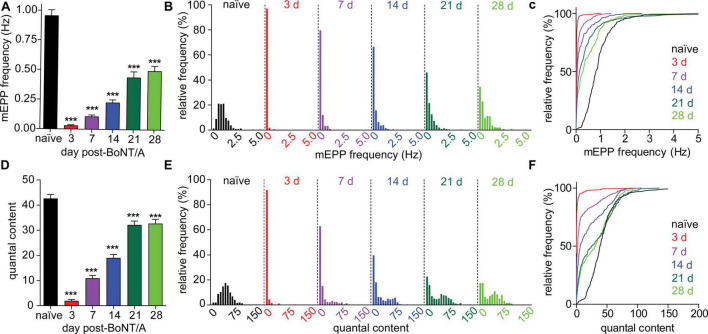
Evoked and spontaneous synaptic transmission recover at different rates following BoNT/A intoxication. Evoked and spontaneous neuromuscular synaptic transmissions were recorded from diaphragms removed from naïve mice and strong responders at 3, 7, 14, and 21, and 28 days after IP injection of 0.7 LD_50_ BoNT/A. **(A)** mEPP frequencies are significantly decreased at 3, 7, 14, 21, and 28 days after BoNT/A injection. Unimodal recovery of mEPP frequencies is apparent in frequency histograms **(B)** as well as cumulative frequency distributions **(C)**. **(D)** QC was reduced at 3, 7, 14, 21, and 28 days after BoNT/A injection. Average mEPP amplitudes from each diaphragm were used to estimate quantal content of end plates producing EPPs but no mEPPs. A bimodal distribution of QC values during recovery is apparent in frequency histograms **(E)** as well as cumulative frequency distributions **(F)**. Arrows indicate populations in which recovery of QC and mEPP frequencies is uncoupled. For naïve mice, *N* = 7 and 249 end plates; for 3 days post-BoNT/A, *N* = 7 mice and 278 end plates; for 7 days post-BoNT/A, *N* = 7 mice and 323 end plates; for 14 days post-BoNT/A, *N* = 6 mice and 297 end plates; for 21 days post-BoNT/A, *N* = 7 mice and 355 end plates; and for 28 days post-BoNT/A, *N* = 6 mice and 256 end plates. Data is plotted as mean and S.D. ***p < 0.001. Statistical significance vs. naïve was determined using the Kruskal-Wallis test with Dunn’s post-test.

To understand the hysteresis between neurophysiological recovery (>28 days) and functional recovery (<21 days) in strong responders, we measured two fundamental parameters of neurotransmission that directly reflect the degree of motor neuron intoxication: miniature EPP (mEPP) frequency and quantal content (QC) (Del Castillo and Katz, 1954). Because spontaneous release is a stochastic phenomenon directly correlated with the availability of functional release complexes, mEPP frequency serves as an agnostic marker of active site availability in intoxicated nerve terminals (Beske et al., 2016; Bradford et al., 2018). Alternatively, QC approximates the number of quanta that fuse during an action potential and thus represents the average release competency during evoked release. The mEPP frequency decreased to 3.2 ± 9.5% of the naïve value at 3 days after intoxication and partially recovered to 52.0 ± 65.3% at 28 days (Figures 4A–C). In comparison, QC was similarly reduced to 4.6 ± 20.1% of naïve values at 3 days but recovered to 76.6 ± 64.0% of naïve end plates at 28 days. Both metrics remained significantly depressed vs. naïve endplates; however, QC recovery was significantly improved compared to mEPP frequency at 28 days (*p* < 0.0001; Figures 4D–F). Consistent with accelerated recovery, QC histograms contained a mixture of recovered and stubbornly impaired subpopulations at 7, 14, 21, and 28 days while mEPP frequency histograms were unimodal until 28 days (Figures 4B, E). Although quantal size declined by 33.7 ± 38.3% within 3 days and remained significantly depressed through 28 days (Supplementary Figures 3C, D), quantal size underwent a monotonic recovery. Consequently, the bimodal recovery of EPP amplitude and QC cannot be attributed to changes in quantal size.

**FIGURE 5:**
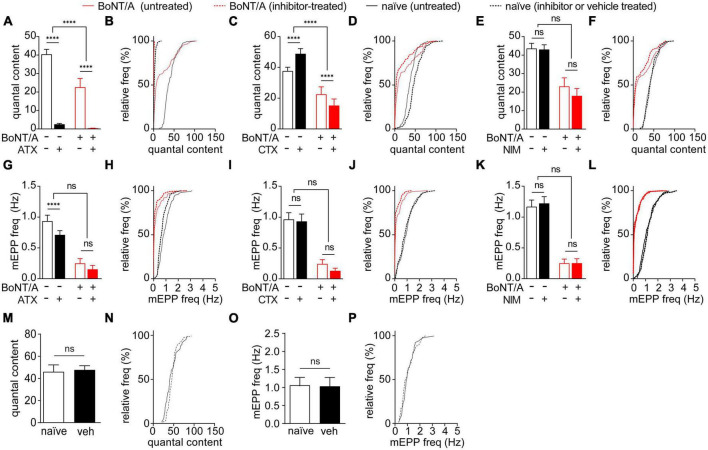
N-type and P/Q-type VGCC activity promotes synaptic transmission in endplates of recovering muscle fibers. The effects of selective VGCC antagonists or vehicle on EPPs and mEPPs were tested in diaphragms removed from naïve mice and strong responders at 21 days after IP injection of 0.7 LD_50_ BoNT/A or saline. **(A,B)** P/Q-type VGCC blocker ATX reduced QC in both naïve end plates and BoNT/A-intoxicated end plates, with a larger effect on BoNT/A-intoxicated end plates. **(C,D)** N-type VGCC blocker CTX increased the QC of naïve end plates but reduced the QC in BoNT/A-intoxicated. Cumulative frequency distributions indicate the greatest inhibition occurred at end plates with the largest QC values. **(E,F)** The L-type VGCC blocker NIM had no effect on QC in naïve end plates or BoNT/A-intoxicated end plates. **(G,H)** Vehicle-treatment of naïve controls did not have a significant effect on QC (*p* = 0.21) **(I–N)** mEPP frequencies between naïve end plates and BoNT/A-intoxicated end plates were not affected by treatment with ATX [**(I,J)**; *p* = 0.81], CTX [**(K,L)**; *p* = 0.14], or NIM [**(M,N)**; *p* = 0.50]. **(O,P)** Vehicle-treatment of naïve controls did not have a significant effect on mEPP frequency (*p* = 0.60). *N* = 6 animals and 111–123 end plates for VGCC blocker-treated groups; *n* = 3 animals and 54–59 end plates for vehicle-treated controls. Data is plotted as mean ± SD of end plate values. ^****^p < 0.0001. The significance of the effect of the VGCC treatment within and between the naïve and BoNT/A-injected groups was determined by two-way ANOVA with Sidak’s multiple comparison post-tests. Statistical significance of vehicle-treated controls was determined using the Mann-Whitney test.

Evoked release is tightly coupled to voltage-gated calcium channel (VGCC) activation. In contrast, mEPPs can be triggered by several mechanisms, including release of internal calcium stores, calcium-regulated signaling or local activation of individual VGCCs (Williams and Smith, 2018; Lee et al., 2022). This raised the possibility that differential regulation of VGCCs contributes to accelerated recovery of QC vs. mEPP frequency. To test this hypothesis, QC and mEPP frequency were compared between diaphragms treated with vehicle or selective antagonists of P/Q-type (ω-agatoxin IVA, ATX), N-type (ω-conotoxin GVIA, CTX) or L-type VGCCs (nimodipine, NIM) (Sano et al., 1987; Protti and Uchitel, 1993; Flink and Atchison, 2002). Selective antagonists were tested on naïve diaphragms and diaphragms prepared 21 days after intoxication, when the difference between recovery of mEPP and QC was largest (Figure 4). Whereas block of P/Q-type VGCC reduced QC under all conditions, the effect was larger in intoxicated end plates (1.6 ± 2.5% of vehicle) than naïve end plates (6.3 ± 6.9% of vehicle; *p* < 0.0001), suggesting P/Q-type VGCCs play a larger role in intoxicated end plates (Figures 5A, B). Block of N-type VGCC had context-specific effects on evoked release, increasing QC to 129.9 ± 49.1% in naïve end plates but decreasing QC to 67.5 ± 102.8% in intoxicated end plates (Figures 5C, D). Block of L-type VGCC had no effect on QC in naïve or intoxicated end plates (*p* ≥ 0.21, Figures 5E, F). Selective VGCC antagonists were also tested on mEPP frequencies. Although block of P/Q-type VGCC reduced mEPP frequency in naïve end plates, the effect was small and not significant between naïve vs. intoxicated end plates (Figures 5G, H). Block of N-type (Figures 5I, J) and L-type (Figures 5K, L) VGCCs did not significantly alter mEPP frequencies in naïve or intoxicated end plates. Control studies showed vehicle treatment did not affect QC or mEPP frequencies in naïve end plates (Figures 5M–P). Taken together, these data indicate that P/Q and N-type VGCCs increase evoked release but not spontaneous release in intoxicated end plates.

## 4. Discussion

The inherent difficulty of studying functional responses of respiratory muscles to BoNT intoxication has limited research into the neurophysiological mechanisms involved in recovery from paralysis. Here, we establish a model of botulism involving respiratory toxemia with low mortality. Recovery of respiratory function was monitored by measuring toxic signs (abdominal paradox and respiratory pattern), running activity, diaphragm function, and synaptic physiology at multiple time points after challenge with doses of BoNT/A that produce a high incidence of toxic signs with low mortality.

Despite the relatively low dose (compared to intramuscular challenges), we found a relatively large portion of intoxicated endplates remained impaired 28 days after intoxication. Recovery of BoNT/A-intoxicated muscles involves extensive growth of new endplate sprouts that are capable of synaptic transmission unlike the original parent endplate (De Paiva et al., 1999). However, rapid endplate outgrowth after intoxication occurs only in slow twitch fibers, and synaptic sprouts take weeks to develop in fast twitch fibers (Duchen, 1971). Diaphragm muscles consist of a mix of slow twitch and fast twitch muscle fibers (Polla et al., 2004), suggesting that stubbornly intoxicated fibers might be fast twitch fibers that do not develop outgrowths until weeks after intoxication.

The most striking finding in these studies was the enhanced recovery of evoked release compared to spontaneous release, suggesting compensatory responses to neuromuscular paralysis that enhanced respiratory function despite prolonged inhibition of synaptic transmission. Several forms of synaptic plasticity were identified that likely contribute to these compensatory responses, including differential recruitment of VGCCs, post-synaptic modulation of resting membrane potential and changes in quantal size. These studies point to potential therapeutic targets and help explain the effectiveness of proposed treatments that directly or indirectly enhance VGCC activity (Adler et al., 1996; Bradford et al., 2018; Vazquez-Cintron et al., 2020; Machamer et al., 2022).

Because this study was designed to characterize the progression of neuromuscular function recovery in animals displaying respiratory signs of botulism, the cutoff for inclusion in the strong responder group was 3 days, which was the first time point chosen to quantify diaphragm function using *ex vivo* assays. However, toxic signs resolved between 3 and 5 days in approximately 50% of strong responders whereas the remaining 50% resolved after 10 days. This raises the possibility that restricting studies to mice that exhibit toxic signs beyond 5 days would further improve the characterization of compensatory changes during recovery. The variable duration of toxic signs is consistent with clinical data (Sobel, 2005), while evidence of long-lasting neurophysiological deficits without associated physiological signs is consistent with single fiber electrode recordings in clinical patients given therapeutic doses of BoNT by intramuscular injection (Kouyoumdjian et al., 2020).

The key mechanistic difference between evoked release and spontaneous release is the identity of the vesicle fusion trigger and the associated proteins. Under physiological conditions, evoked release is primarily triggered by Ca^2+^ binding to synaptotagmin-1 and synaptotagmin-7 in response to action potential (AP) induced activation of VGCCs (Rizo and Rosenmund, 2008; Jahn and Fasshauer, 2012). Conversely, spontaneous vesicle fusion is loosely coupled to Ca^2+^ influx and the identity of the Ca^2+^ sensor and the mechanisms driving vesicle fusion remain uncertain (Xu et al., 2009; Groffen et al., 2010; Kavalali, 2015). AP-induced increases in cytosolic Ca^2+^ transients can potentiate spontaneous vesicle fusion, but with substantially lower sensitivity than in evoked release (Hubbard et al., 1968; Beske et al., 2017), and activity-dependent potentiation of VGCCs increases evoked release, while having little effect on spontaneous release (Frank et al., 2006; Jeans et al., 2017; Böhme et al., 2018). Thus, enhancement of AP-induced VGCC Ca^2+^ currents could contribute to the accelerated recovery of evoked release without significantly affecting spontaneous release.

Although the C-terminal nine residues cleaved by BoNT/A (producing SNAP-25_197_) are not required for association of SNAP-25 with ternary SNARE complexes (Banerjee et al., 1996), their cleavage destabilizes the SNARE complex and reduces the efficiency of the fusogenic configuration, thereby inhibiting vesicle release (Huang et al., 1998). Indeed, release complexes containing SNAP-25_197_ remain competent to effect vesicle fusion, but only at elevated Ca^2+^ concentrations (Trudeau et al., 1998; Weber et al., 2010; Beske et al., 2017). Thus, it is possible that vesicles associated with full-length SNAP-25 but not SNAP-25_197_ contribute to spontaneous fusion events, whereas the increased Ca^2+^ levels associated with evoked release are required to recruit SNAP-25_197_ (Beske et al., 2018), thus contributing to the difference in recovery between evoked release and spontaneous release in motor neurons intoxicated by BoNT/A.

Vesicle fusion is mediated by three high voltage-activated VGCCs distinguished by the alpha subunit (Dolphin, 2016): P/Q-type (CaV2.1), L-type (CaV1), and N-type (CaV2.2). Under physiological conditions, P/Q-type VGCCs are the principal VGCC used to trigger vesicle fusion in adult mammalian motor neurons. However, during the early stages of development (Rosato Siri and Uchitel, 1999), or after physical, chemical or genetic, synaptic transmission becomes sensitive to N-type, L-type, and R-type channel blockers injury (Katz et al., 1996; Fratantoni et al., 2000; Urbano et al., 2001, 2003; Flink and Atchison, 2002). We found inhibition of P/Q-type and N-type channel activity decreases evoked release in intoxicated diaphragms, indicating that intoxicated endplates are more sensitive to the loss of P/Q- and N-type VGCCs and suggesting that a homeostatic increase in P/Q-type and N-type channel activity can promote evoked release in BoNT/A-intoxicated motor neurons. Indeed, selective VGCC recruitment in intoxicated NMJs may contribute to the strong effects of aminopyridines and selective VGCC agonists at BoNT-intoxicated synapses (Beske et al., 2017, 2018; Bradford et al., 2018). Similar homeostatic increases in synaptic transmission occur in response to postsynaptic receptor blockade and are mediated by increased P/Q-type VGCC currents (Wang et al., 2004; Jeans et al., 2017). Although the mechanisms underlying homeostatic compensatory increases in QC in mammalian NMJs have not been fully characterized (Wang et al., 2016, 2018), numerous studies have identified mechanisms of homeostatic QC increases at the Drosophila NMJ (Dickman et al., 2012; Frank, 2014). In contrast to the loss of synaptic vesicle fusion in BoNT/A intoxicated endplates, most of these studies used pharmacological or genetic methods to inhibit synaptic transmission (DiAntonio et al., 1999; Frank et al., 2006). However, reducing presynaptic innervation (Davis and Goodman, 1998) or muscle excitability is sufficient to induce compensatory increases in quantal content (Paradis et al., 2001). Several retrograde signaling pathways communicate the loss of postsynaptic function (Haghighi et al., 2003; McCabe et al., 2003; Goold and Davis, 2007; Frank et al., 2009), many of which converge to increase calcium flux through Cacophony (Frank et al., 2006, 2009), the Drosophila homolog of mammalian N and P/Q-type VGCCs (Smith et al., 1996; Brooks et al., 2003), or increase coupling between Cacophony and the synaptic vesicle release machinery (Frank et al., 2020). Other homeostatic mechanisms may also contribute to compensatory plasticity; for example, it has been shown in primary rat glutamatergic neurons that knock-down of SNAP-25 increases VGCC current density without affecting VGCC subtype proportions or the expression or localization of P/Q-type channels (Condliffe et al., 2010). Thus, although BoNT/A is not known to change the level of expression or localization of SNAP-25, it is possible that cleavage of SNAP-25 alters regulation of VGCC function.

Blockade of N-type channels has previously been reported to have no effect on neuromuscular synaptic transmission (Wright and Angus, 1996; Santafé et al., 2000; Giovannini et al., 2002). This discrepancy with our data could indicate a small effect size, such that contributions of N-type channels to neurotransmission are only detectable in experiments with many recordings, such as conducted here. Alternatively, the discrepancy might be attributable to divergent animal behavior because of differential housing conditions. Naïve mice in our study were given access to free-spinning running wheels for 12 days prior to experimentation. It has been shown that mice housed with running wheels have increased rates of exercise, respiration and diaphragm activity (Tallis et al., 2017), which can elicit activity-dependent changes in motor neuron synaptic structure and function (Nishimune et al., 2014). Given that N-type channel activity both promotes synaptic transmission in BoNT/A-paralyzed diaphragms and inhibits synaptic transmission in diaphragms from exercising mice, N-type channels may have conditional effects at the neuromuscular junction that extend beyond directly promoting vesicle fusion, such as regulating bidirectional activity-dependent synaptic plasticity. Indeed, Ca^2+^ signaling through VGCCs is an essential component of bidirectional synaptic plasticity in motor neurons (Liang et al., 2003; Catterall and Few, 2008), and N-type channels could differentially regulate synaptic transmission by initiating Ca^2+^ signaling cascades that modify release downstream of P/Q channel activity. Although N-type channels are present at rodent NMJs, they appear to be expressed in the terminal Schwann cell (TSC), a glial cell covering the presynaptic terminal, and not in motor neurons (Day et al., 1997). The function of TSCs has primarily been characterized during neuromuscular reinnervation following nerve injury (Barik et al., 2016; Santosa et al., 2018). However, TSCs also mediate bidirectional plasticity based on the characteristics of cytoplasmic Ca^2+^ waves (Todd et al., 2010), consistent with the observation that VGCCs are expressed in many types of Glia including schwann cells, and VGCC-dependent calcium signaling in Glia is essential for maintaining changes in synaptic efficacy (Verkhratsky et al., 1998; Fiacco and McCarthy, 2006; Letellier et al., 2016). Future work should directly test the hypothesis that N-type channels contribute to bidirectional activity-dependent plasticity by modulating Ca^2+^ signaling in TSCs.

Consistent with other models of BoNT/A intoxication (Sellin and Thesleff, 1981; Kim et al., 1984; Molgó et al., 1989), we observed a decrease in mEPP amplitudes in diaphragm muscle fibers 3 days after BoNT injection, followed by a partial recovery through 28 days. Recovery of mEPP amplitude could enhance muscle fiber function by reducing the number of quanta required to trigger muscle contraction. Postsynaptic changes in receptor activity and localization are believed to be the primary locus of quantal size regulation (Turrigiano and Nelson, 2004), but multiple presynaptic mechanisms regulating mEPP amplitude have also been identified (Wang et al., 2005; Fong et al., 2010; Goh et al., 2011; Morsch et al., 2018). Pharmacologically targeting these regulatory pathways to increase mEPP amplitude is anticipated to promote motor neuron recovery, especially in stubbornly impaired end plates. Although inhibiting acetylcholinesterase in the synaptic cleft has been shown clinically to promote muscle function in botulism patients (Young and Halstead, 2014; Boerner et al., 2018), we are unaware of any studies focusing on modulation of mEPP amplitudes to treat botulism. Recently, compounds targeting presynaptic cannabinoid receptors have been found to regulate mEPP amplitudes and have been shown effective at restoring neuromuscular transmission in myasthenic mice (Morsch et al., 2018), suggesting they may have therapeutic potential for treating botulism.

We observed a depolarizing shift in muscle fiber RMP following intoxication, consistent with other studies investigating the loss of synaptic transmission as a consequence of BoNT intoxication (Colméus et al., 1982) or denervation (Mathers and Thesleff, 1978; Bray et al., 1982). The RMP is set by the permeability of K^+^ channels, the activity of Na^+^/K^+^ pumps, and to a lesser extent the permeability of Na^+^ and Cl^–^ channels (Fauler et al., 2012). Multiple types of disuse atrophy share a common transcriptional response (Lecker et al., 2004; Sacheck et al., 2007), and long-term disuse upregulates voltage-dependent Na^+^ and ClC-1 chloride channels (Desaphy et al., 2001; Pierno et al., 2002) and down-regulates the ATP-sensitive K^+^ channels (Tricarico et al., 2010). Similar changes likely occur because of motor neuron paralysis from BoNT intoxication. However, among the few studies that have identified changes in postsynaptic transcriptional programming after BoNT intoxication, none have identified changes in gene expression that directly regulate RMP (Shen et al., 2006; Mukund et al., 2014). Considering the speed of RMP reduction after BoNT/A intoxication, local signaling mechanisms that are independent from transcription may contribute to changes in RMP. In denervated rat diaphragms, RMP is modulated by the loss of glutamatergic and cholinergic anterograde signaling (Urazaev et al., 1995, 1997; Proskurina et al., 2018), and recent work has identified a pathway by which AChRs directly modify RMP through altering Na,K-ATPase pump activity (Krivoi et al., 2006; Heiny et al., 2010; Kravtsova et al., 2016). More work is needed to determine the functional consequences of these changes on muscle excitability. However, depolarization of the muscle fiber membrane at voltages closer to the voltage-gated Na^+^ channel threshold increases its intrinsic excitability. These studies suggest the mechanisms regulating RMP after BoNT intoxication could be multifaceted and evolve during recovery. The large number of druggable membrane proteins involved in regulating membrane potential offer fertile targets for pharmacological interventions to promote muscle contraction.

In conclusion, we describe a reproducible, sublethal model of botulism well-suited for evaluating post-symptomatic pharmacotherapies aimed at enhancing the recovery of respiratory function. We identified multiple forms of endogenous plasticity that compensate for low-amplitude synaptic transmission in diaphragm NMJs. These compensatory mechanisms exploit the malleability of release probability, quantal size and membrane potential of muscle fibers to drive membrane potential closer to muscle contraction threshold during evoked synaptic transmission. The effectiveness of these endogenous mechanisms is demonstrated by the rapid recovery of diaphragm function, respiration, and motility despite a subpopulation of diaphragm end plates that remain stubbornly impaired. Treatments that synergize with these changes could be effective at all stages of botulism.

## Data availability statement

The raw data supporting the conclusions of this article will be made available by the authors, without undue reservation.

## Ethics statement

The experimental protocol was approved by the United States Army Medical Research Institute of Chemical Defense (USAMRICD) IACUC. This study was conducted in accordance with the local legislation and institutional requirements.

## Author contributions

JM, EV-C, MS, KP, AB, CO, and PM contributed to conception, design, and execution of the study. JM, EV-C, and PM wrote sections of the manuscript. All authors contributed to manuscript revision, read, and approved the submitted version.

## Supplementary material

Supplementary Figure 1 Determination of BoNT/A potency in female CD1 mice. Mice were challenged by intraperitoneal injection with 0.10, 0.125, 0.150, 0.175, 0.20, 0.225, 0.250, or 0.30 ng/kg BoNT/A (*n* = 4–8 per group) and survival rates were monitored over 4 days. Surviving mice are depicted in black; deceased mice are depicted in red. The median lethal dose was estimated to be 0.17 ng/kg (95% CI: 0.14–0.20 ng/kg) from simple logistical regression of survival outcomes.

Supplementary Figure 2 Behavioral correlates of intoxication and recovery from sublethal systemic botulism. **(A)** Exemplar traces of running wheel activity of a strong responder after intraperitoneal challenge with 0.7 LD_50_ BoNT/A (day of injection indicated by red trace) and **(B)** total distance run per night prior to BoNT/A injection (red dot) and the 20 days following injection. **(C)** Exemplar traces of running wheel activity of a weak responder after BoNT/A injection and **(D)** total distance run per night. **(E)** Exemplar traces of running wheel activity of a mouse injected with BoNT/A followed by equimolar sheep anti-BoNT/A antitoxin and **(F)** total distance run per night. Compared to baseline running activity, strong responders (but not weak responders or antitoxin-treated mice) ran less per night **(G)**, less per 5 min interval **(H)**, and fewer 5 min intervals per night **(I)** after intoxication [*N* = 32–36 for strong responders (4 mice were removed for muscle contraction experiments during recovery), *N* = 24 for weak responders, and *N* = 8 for antitoxin-treated mice]. Due to overlapping data points, recovery data are presented as mean with S.E.M for clarity and interpretability. Dots indicate statistical significance compared to baseline measurements of indicated colored trace. ^***^*p* < 0.001, **p* < 0.05. Absence of dots indicates that values were not statistically significant. Statistical significance was determined by two-way RM ANOVA and Bonferroni’s multiple comparison post-tests comparing each time point to the day prior to injection.

Supplementary Figure 3 Intoxication has persistent effects on RMP and quantal size. **(A)** Muscle fiber RMPs were reduced at 3, 7, and 14 days after intoxication. **(B)** Cumulative frequency distribution indicates a shift of abnormally distributed RMP population after intoxication. **(C)** mEPP amplitudes were reduced at 3, 7, 14, 21, and 28 days after intoxication. **(D)** Cumulative frequency distributions reveal a skewed shift of normally distributed mEPP amplitudes after intoxication. For RMP: *N* = 7 naïve mice and 249 end plates; for 3 days post-BoNT/A, *N* = 7 mice and 278 end plates; for 7 days post-BoNT/A, *N* = 7 mice and 323 end plates; for 14 days post-BoNT/A, *N* = 6 mice and 297 end plates; for 21 days post-BoNT/A, *N* = 7 mice and 355 end plates; and for 28 days post-BoNT/A, *N* = 6 mice and 256 end plates. For some end plates, mEPP recordings were unsuccessful or no mEPPs were detected. Consequently, for mEPP amplitude: *N* = 7 naïve mice and 235 end plates; for 3 days post-BoNT/A, *N* = 7 mice and 208 end plates; for 7 days post-BoNT/A, *N* = 7 mice and 279 end plates; for 14 days post-BoNT/A, *N* = 6 mice and 268 end plates; for 21 days post-BoNT/A, *N* = 7 mice and 336 end plates; and for 28 days post-BoNT/A, *N* = 6 mice and 228 end plates. Data are plotted as mean and S.D. ^***^*p* < 0.001, ^**^*p* < 0.01. Statistical significance vs. naïve end plates was determined using the Kruskal-Wallis test with Dunn’s post-test for success rates and EPP amplitudes and using one-way ANOVA with Dunnett’s post-test for mEPP amplitudes and RMPs.
