# Supplementary figures and images for: Neuromuscular recovery from botulism involves multiple forms of compensatory plasticity
Source: Front Cell Neurosci. 2023 Aug 15;17:1226194. doi: 10.3389/fncel.2023.1226194 (PMC10463753; doi:10.3389/fncel.2023.1226194)

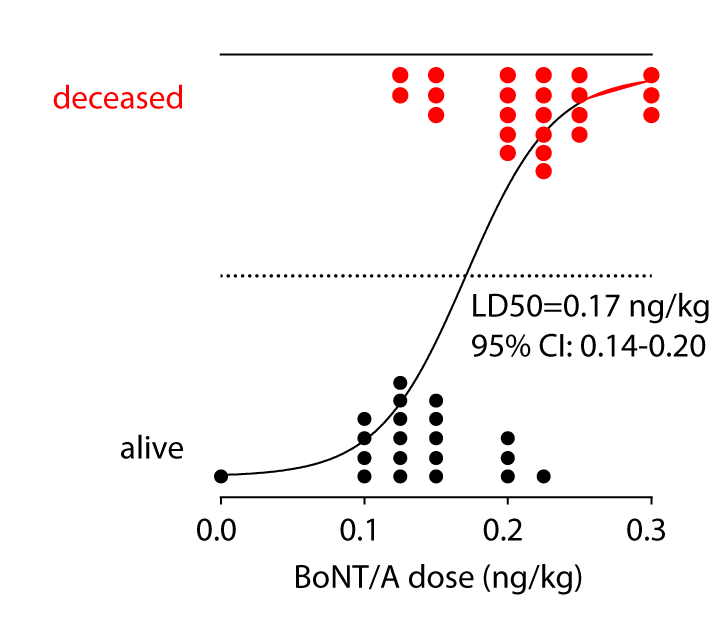

Supplement: Supplementary Figure 1 — Determination of BoNT/A potency in female CD1 mice. Mice were challenged by intraperitoneal injection with 0.10, 0.125, 0.150, 0.175, 0.20, 0.225, 0.250, or 0.30 ng/kg BoNT/A (n = 4–8 per group) and survival rates were monitored over 4 days. Surviving mice are depicted in black; deceased mice are depicted in red. The median lethal dose was estimated to be 0.17 ng/kg (95% CI: 0.14–0.20 ng/kg) from simple logistical regression of survival outcomes. [file Image_1.TIF]

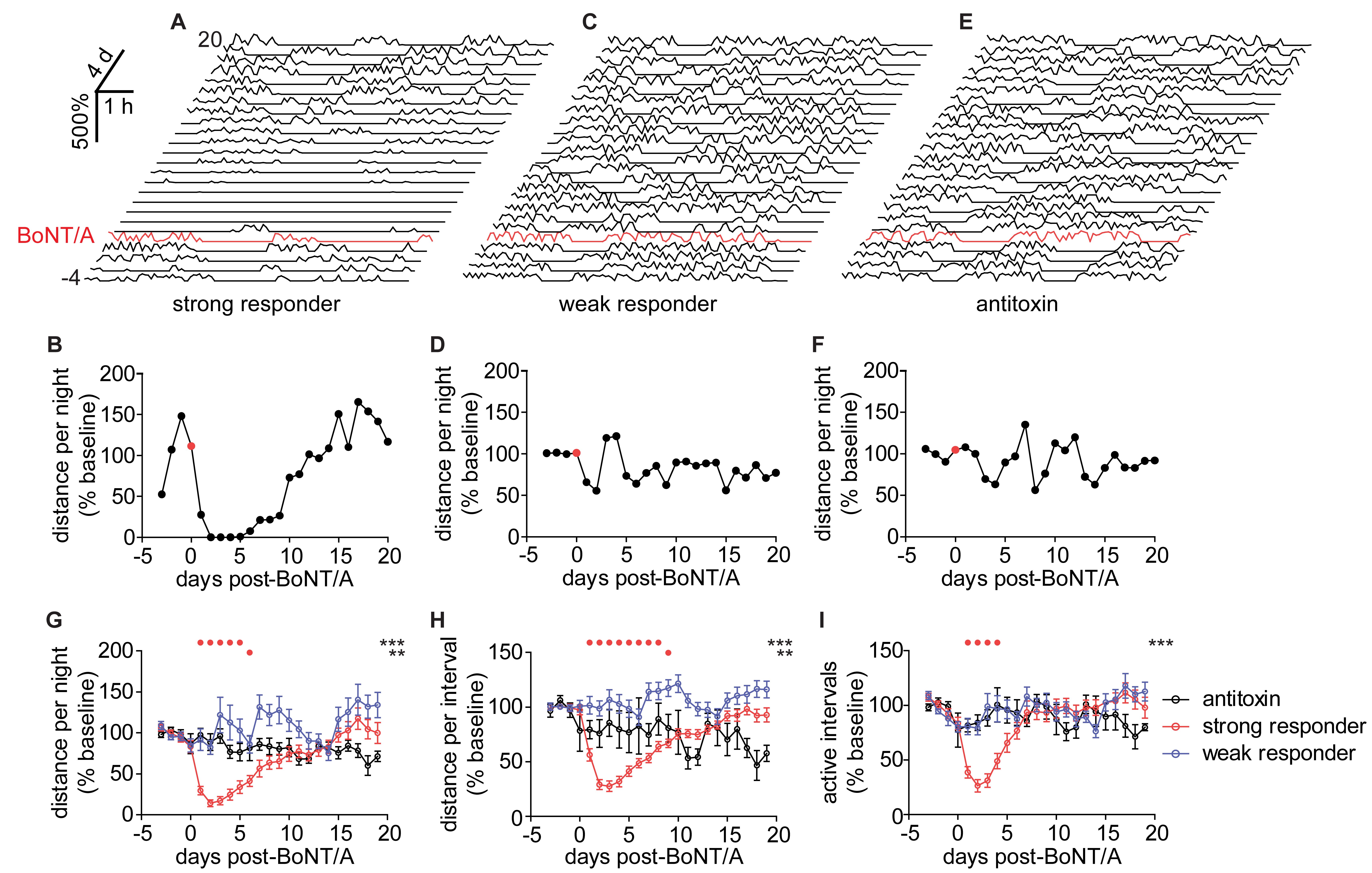

Supplement: Supplementary Figure 2 — Behavioral correlates of intoxication and recovery from sublethal systemic botulism. (A) Exemplar traces of running wheel activity of a strong responder after intraperitoneal challenge with 0.7 LD50 BoNT/A (day of injection indicated by red trace) and (B) total distance run per night prior to BoNT/A injection (red dot) and the 20 days following injection. (C) Exemplar traces of running wheel activity of a weak responder after BoNT/A injection and (D) total distance run per night. (E) Exemplar traces of running wheel activity of a mouse injected with BoNT/A followed by equimolar sheep anti-BoNT/A antitoxin and (F) total distance run per night. Compared to baseline running activity, strong responders (but not weak responders or antitoxin-treated mice) ran less per night (G), less per 5 min interval (H), and fewer 5 min intervals per night (I) after intoxication [N = 32–36 for strong responders (4 mice were removed for muscle contraction experiments during recovery), N = 24 for weak responders, and N = 8 for antitoxin-treated mice]. Due to overlapping data points, recovery data are presented as mean with S.E.M for clarity and interpretability. Dots indicate statistical significance compared to baseline measurements of indicated colored trace. ***p < 0.001, *p < 0.05. Absence of dots indicates that values were not statistically significant. Statistical significance was determined by two-way RM ANOVA and Bonferroni’s multiple comparison post-tests comparing each time point to the day prior to injection. [file Image_2.tif]

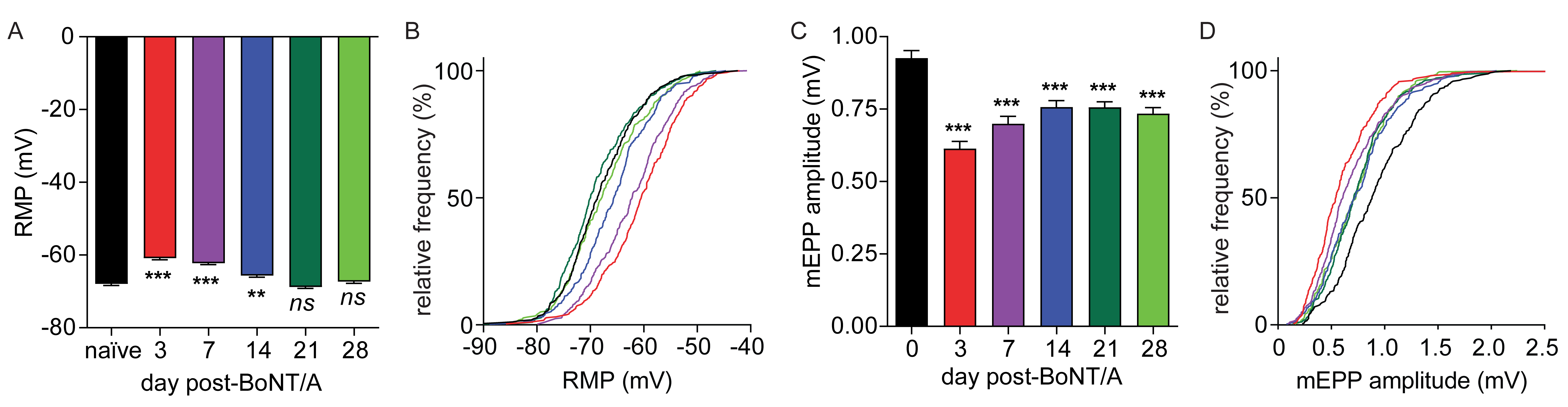

Supplement: Supplementary Figure 3 — Intoxication has persistent effects on RMP and quantal size. (A) Muscle fiber RMPs were reduced at 3, 7, and 14 days after intoxication. (B) Cumulative frequency distribution indicates a shift of abnormally distributed RMP population after intoxication. (C) mEPP amplitudes were reduced at 3, 7, 14, 21, and 28 days after intoxication. (D) Cumulative frequency distributions reveal a skewed shift of normally distributed mEPP amplitudes after intoxication. For RMP: N = 7 naïve mice and 249 end plates; for 3 days post-BoNT/A, N = 7 mice and 278 end plates; for 7 days post-BoNT/A, N = 7 mice and 323 end plates; for 14 days post-BoNT/A, N = 6 mice and 297 end plates; for 21 days post-BoNT/A, N = 7 mice and 355 end plates; and for 28 days post-BoNT/A, N = 6 mice and 256 end plates. For some end plates, mEPP recordings were unsuccessful or no mEPPs were detected. Consequently, for mEPP amplitude: N = 7 naïve mice and 235 end plates; for 3 days post-BoNT/A, N = 7 mice and 208 end plates; for 7 days post-BoNT/A, N = 7 mice and 279 end plates; for 14 days post-BoNT/A, N = 6 mice and 268 end plates; for 21 days post-BoNT/A, N = 7 mice and 336 end plates; and for 28 days post-BoNT/A, N = 6 mice and 228 end plates. Data are plotted as mean and S.D. ***p < 0.001, **p < 0.01. Statistical significance vs. naïve end plates was determined using the Kruskal-Wallis test with Dunn’s post-test for success rates and EPP amplitudes and using one-way ANOVA with Dunnett’s post-test for mEPP amplitudes and RMPs. [file Image_3.TIF]
